# TRPV4/NCX1 coupling regulates Ca^2+^‐dependent cholecystokinin secretion from enteroendocrine cells to involve the pathogenesis of irritable bowel syndrome‐diarrhea in male mice

**DOI:** 10.14814/phy2.71149

**Published:** 2026-10-20

**Authors:** Jianxin Wang, Xiongying Chen, Shuo Tong, Chengxin Qu, Wei Lu, Hui Dong

**Affiliations:** ^1^ Department of Pharmacology, School of Pharmacy Qingdao University Medical College Qingdao China; ^2^ Department of Pediatrics Guangyuan Central Hospital Guangyuan Sichuan China

**Keywords:** cholecystokinin, enteroendocrine cells, irritable bowel syndrome‐diarrhea, NCX1, TRPV4 channels

## Abstract

Although stress can stimulate the vagus nerve via the brain–gut axis to induce enterohormone secretion from enteroendocrine cells (EECs), little is currently known about the roles of ion channels in the regulation of enterohormone secretion and the development of irritable bowel syndrome (IBS). We aimed to investigate whether transient receptor potential vanilloid 4 (TRPV4) channels and Na^+^/Ca^2+^ exchanger 1 (NCX1) are critical for enterohormone secretion and whether they are involved in IBS. Ca^2+^imaging and patch‐clamp were applied to STC‐1. IBS‐diarrhea (IBS‐D) mouse model was established by wrap restraint stress (WRS). Western blot verified TRPV4 and NCX1 expressions in STC‐1; co‐immunoprecipitation demonstrated their physical interaction, and Ca^2+^ imaging and patch‐clamp confirmed function and interaction. Carbachol (CCh), a stable analog of acetylcholine (ACh), induced Ca^2+^ signaling and currents in EECs via a functional coupling of TRPV4 and NCX1. Moreover, intestinal cholecystokinin (CCK) transcripts in IBS‐D mice were significantly increased, and ACh enhanced intestinal CCK secretion in mice, all of which could be abolished by blockers of TRPV4, NCX1, and CCK receptors. Finally, these blockers alleviated the severity of IBS‐D in mice. We reveal a novel TRPV4/NCX1/Ca^2+^/CCK pathway in EECs, which is involved in the pathogenesis of IBS‐D. Targeting this pathway may hold promise for the prevention/treatment of IBS‐D.

## Graphical abstract

ACh regulates the increase of intracellular Ca^2+^ signaling through TRPV4/NCX1 coupling, inducing CCK release, and participates in the pathogenesis of IBS‐D.

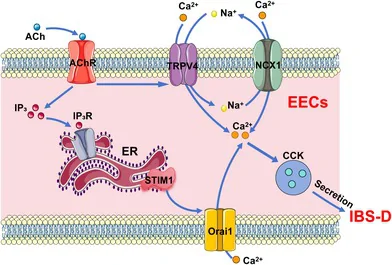

## 1 INTRODUCTION

Irritable bowel syndrome (IBS) is one of the most prevalent chronic functional gastrointestinal (GI) disorders in humans, characterized primarily by abdominal pain, bloating, and alterations in bowel habits without detectable evidence of organic lesions in the intestine (Altomare et al., 2021). Due to predominant symptoms, IBS is divided into four groups: predominant constipation (IBS‐C), predominant diarrhea (IBS‐D), mixed bowel habits (IBS‐M), and the predominant symptoms are unclassified (IBS‐U) (Bonetto et al., 2021). The pathogenesis of IBS may involve intestinal flora, dietary habits, gastrointestinal motility, visceral hypersensitivity, and some other factors (Alammar & Stein, 2019; Fukudo & Kanazawa, 2011; Noemi et al., 2024). IBS is also considered a disorder of gut‐brain interaction since psychological stress significantly impacts the occurrence and progression of IBS, especially in IBS‐D (Ancona et al., 2021; Ford et al., 2020). However, little is known about the molecular mechanisms of the brain–gut axis involved in IBS. A comprehensive understanding of the etiology and pathogenesis of IBS has not yet been achieved due to its intricate etiology, which has greatly hindered the prevention and therapy for IBS. Therefore, it is urgent to further investigate the molecular pathogenesis of IBS.

Recent studies show that the vagus nerve in IBS patients is usually under an hyperexcitable state, and therefore the neurotransmitter acetylcholine (ACh) released from the excited vagus nerve may play an important role in the pathogenesis of IBS (Feng et al., 2024; Ishioh et al., 2024). The vagus nerve modulates intestinal hormone secretion from enteroendocrine cells (EECs) via cholinergic signaling, a pivotal component of the brain–gut axis (Bonaz et al., 2018); however, the regulatory mechanisms are largely unknown. It is well established that enterohormones are crucial in maintaining gastrointestinal (GI) homeostasis (Gribble & Reimann, 2019; Woźniak et al., 2021), and that the abnormality in enterohormone secretion is closely related to the pathogenesis of several GI disorders, such as IBS (Li & Li, 2007). In IBS patients, changes in enterohormone secretion are observed, both in the fasting state and postprandially. Although the role of enterohormones in GI disease seems to be relatively established, some questions still need to be clearly answered, such as the close association of enterohormone cholecystokinin (CCK) and IBS (Qin et al., 2020; Zhen et al., 2021). The increased CCK release after a meal may be involved in the exaggerated postprandial colonic motor response in IBS patients and appears to be abnormally prolonged (Qin et al., 2020). However, the current evidence for CCK irregularities in IBS is not conclusive. In addition, although it is established that CCK secretion from EECs is a Ca^2+^‐dependent process, little is known about the roles of Ca^2+^‐permeable channels and transporters in the regulation of CCK secretion from EECs.

Transient receptor potential (TRP) channels are extensively expressed in GI tract (Dvornikova et al., 2024), and their subfamily transient receptor potential vanilloid (TRPV) channels are involved in the pathogenesis role in IBS (Maqoud et al., 2023; Perna et al., 2021; Szymaszkiewicz et al., 2020), such as one of TRPV channel members, TRPV4 channels that exhibit high permeability to Ca^2+^ in intestinal cells (Cui et al., 2021; Du et al., 2016; Goswami, Cohen, et al., 2017; Goswami, Merth, et al., 2017; Sharma et al., 2017). It was found that endogenous TRPV4 activators were elevated in IBS‐D patients to induce abnormal intestinal secretion and visceral hypersensitivity (Cenac et al., 2010, 2015), but TRPV4 channel blockers could alleviate symptoms of visceral pain in IBS‐D patients (McGuire et al., 2018). Although these data suggest a crucial role of TRPV4 channels in pathogenesis of IBS, it is still unclear whether TRPV4 channels regulate CCK secretion from EECs and whether TRPV4‐mediated CCK is involved in the pathogenesis of IBS.

Na^+^/Ca^2+^ exchanger (NCX) plays a pivotal role in the regulation of intracellular Ca^2+^ and Na^+^ concentrations in intestinal cells (Cantero‐Recasens et al., 2019; Khananshvili, 2020; Nishiyama et al., 2016; Zheng et al., 2020). In general, the ion flux of NCX1 consists of three Na^+^ fluxes with one Ca^2+^ flow to the intracellular or extracellular side of cells (Kang & Hilgemann, 2004). Although NCX1 has been demonstrated in the intestine, little is known about its expression and function in EECs. In addition, numerous studies have demonstrated the function of either TRPV4 channels or NCX1 in intestinal cells (Cui et al., 2021), however, it remains elusive whether TRPV4 channels and NCX1 in EECs functionally couple to regulate Ca^2+^‐dependent CCK secretion and whether they are involved in the pathogenesis of IBS.

Therefore, the objective of this study is to explore: (1) molecular mechanisms of cholinergic signaling‐mediated Ca^2+^‐dependent enterohormone secretion from EECs; (2) the impact of TRPV4 channels and NCX1 on Ca^2+^‐dependent CCK secretion from EECs as well as their influence on the phenotype of IBS. In the present study, STC‐1, an intestinal secretion cell line, was used as a stable and reliable model of native EECs (McCarthy et al., 2015) since: (1) STC‐1 cells usually serve as a classic CCK‐secreting cell model, (2) they exhibit numerous characteristics resembling natural EECs, and (3) their secretion is linked to elevated intracellular Ca^2+^ signaling.

## 2 MATERIALS AND METHODS

### 2.1 Cell culture

Endocrine cells in the small intestine of mice, STC‐1 cells were obtained from Cellverse Bioscience (Cat. No. iCell‐m055, iCell Cellverse, Co. China, ShangHai). Culture medium was comprised of Dulbecco's Modified Eagle Medium (DMEM, Cat. No. C11965500BT, Gibco, Co. USA) supplemented with 10% fetal bovine serum (FBS, Cat. No. FSP500, ExCell, Co. China, ShangHai), and 1 vol‰ penicillin/streptomycin (Cat. No. C0222, Beyotime Biotechnology, Co. China). Cells were incubated at 37°C in a humidified atmosphere containing 5% CO_2_, and the medium was changed twice a week. Prior to intracellular Ca^2+^ measurements and electrophysiological recordings, cells were seeded onto 8‐mm coverslips (Cat. No. YA0353, Solarbio, Co. China) to reach the appropriate density for experimentation.

### 2.2 Animals studies and ethics

All animal care and experimental procedures complied with the “Guide for the Care and Use of Laboratory Animals” published by the National Institutes of Health, USA; all the animal experiments were approved by the Ethics Committee of Medical College of Qingdao University (QUMC). Animal studies are reported in compliance with the ARRIVE guidelines (Percie Du Sert et al., 2020). Male C57BL/6 mice (6–8 weeks old, 20–22 g) were obtained from Jinan Pengyue Laboratory Animal Co. Ltd. (Jinan, Co. China) and maintained in a temperature and humidity‐controlled facility (25°C ± 2°C, 50% ± 5% relative humidity) under a 12 h light/dark cycle with unrestricted access to a diet of chow (Shandong Pengyue Laboratory Animal Technology Co., LTD, and the standards it complies with are GB14924.1, GB14924.2, and GB14924.3) and water. After each experiment, mice were killed by cervical dislocation under anesthesia with 100% CO_2_. Animals were assigned randomly to different experimental groups for all studies. The animals were divided into five groups: the control group, the WRS group, the intraperitoneal injection TRPV4 channel blocker HC067047 group, the intraperitoneal injection NCX1 blocker KB‐R7943 group, and the intraperitoneal injection CCK receptor blocker Devazepide group. Data collection and evaluation of all experiments were performed blindly, and the experimenters were unaware of group treatments.

### 2.3 Electrophysiological recordings

Patch‐clamp recording was performed using an EPC‐10 patch‐clamp amplifier and PatchMaster software (HEKA Electronik GmbH) as described before (Sun et al., 2023). Patch pipettes were pulled from borosilicate glass (Cat. No. BF150N86, Biospikes, Co. China) using a horizontal micropipette puller (Cat. No. P97, Sutter Instruments, Co. USA), with resistance of 3–5 MΩ. STC‐1 cells on a coverslip were mounted in a chamber with an extracellular solution containing (in mM): 140 NaCl, 5 KCl, 2 CaCl_2_, 2 MgCl_2_ and 10 HEPES, and pH adjusted to 7.4 with NaOH. The internal pipette solution was composed of (in mM): 140 CsC1, 0.5 EGTA, 3 Mg‐ATP, 10 HEPES, and pH adjusted to 7.2 with NaOH. Recordings were digitized (10 kHz) for storage on a computer and analyzed using PatchMaster software. Cells were clamped at a voltage of 0 mV to inactivate voltage‐gated sodium channels and 100 ms linear ramps from −100 mV to +100 mV were applied every 2 s. Pipette voltages (Vp) are referred to the bath. In the whole‐cell configuration, Vp corresponds to the membrane potential, and upward deflections of the current trace indicate outward membrane current. The mean membrane capacitance (Cm) and mean access resistance (Rs) were calculated and presented using PatchMaster software (HEKA Electronik GmbH). The leakage current was recorded and subsequently subtracted by the HEKA software. Capacitive currents were compensated for through both fast and slow capacitance compensation, as well as series resistance compensation provided by the software. Additionally, the liquid junction potential was calculated and appropriately compensated for by the software. We determine the corresponding current density by calculating the ratio of the measured current to the capacitance value recorded by the amplifier on the membrane. Results are compared with control studies measured on the same day to minimize any effects of day‐to‐day variability and are reported as current. The number of experiments in each group is five.

### 2.4 Fluorescence Ca^2+^ imaging

Cells were cultured for 24 h on 8‐mm coverslips (Cat. No. YA0353, Solarbio, Co. China) and then loaded with 5 μM Fura‐2 (Cat. No. F8460, Solarbio, Co. China) in isotonic extracellular buffer containing (in mM): 140 NaCl, 5 KCl, 2 CaCl_2_, 2 MgCl_2_, 10 Glucose, 10 HEPES (pH 7.4) supplemented with 0.01% pluronic F127 (Cat. No. P6791, Solarbio, Co. China) for 60 min at 37°C. The coverslip was placed in the perfusion chamber on the stage of an inverted fluorescent microscope (Olympus IX73, 20×, Co. Japan). Changes in intracellular Ca^2+^ signaling were measured at excitation wavelengths of 340 nm for calcium‐bound Fura‐2 and 380 nm for calcium‐free Fura‐2 and at emission wavelength of 510 nm. Experiments were performed at room temperature via MetaFluor software (Molecular Devices, Co. USA). Images were captured using a digital camera (Cat. No. C11440‐42U30, HAMAMATSU, Co. Japan). We performed baseline zeroing on all Ca^2+^ imaging data, making the results and conclusions of the relevant experiments clearer and distinct. The number of cells in each group of experiments is ≥10.

### 2.5 RNA extraction, reverse transcriptase reaction and RT‐qPCR

Crushing the intestinal tissue and total RNA was isolated using RNAeasy™ Animal Long RNA Isolation Kit (Cat. No. R0027, Beyotime Biotechnology, Co. China) according to the manufacturer's instructions. RNA concentration was quantified using Nano‐300 Micro‐Spectrophotometer (Cat. No. Nano‐300, ALLSHENG, Co. China). 1 μg of total RNA was reverse‐transcribed at 37°C for 45 min in the presence of 2 μL dNTP Mix (10 mM each) (Cat. No. D7168L, Beyotime Biotechnology, Co. China) and 1 μL BeyoRT™ II M‐MuLV Reverse Transcriptase (Cat. No. D7168L, Beyotime Biotechnology, Co. China). Real‐time quantitative PCR started at 95°C for 30 s, followed by 40 cycles of denaturation at 95°C for 10 s, annealing and extension at 60°C for 30 s and melting curve 65°C–95°C, with 0.5°C increase every 5 s. PCR amplification was carried out using Sybr Green PCR Master Mix (Cat. No. 11202ES08, Yeasen, Co. China) and specific primers for GAPDH and CCK (Qingdao Ruibio Biotech Co., Ltd. Co. China). The relative expression levels of CCK mRNAs were normalized to a reference gene, mGAPDH. RT‐qPCR was repeated three times. The results are calculated using the 2^−ΔΔCt^ methods (Livak & Schmittgen, 2001). Primers used for RT‐qPCR analysis are designed using the online primer‐blast tool (Ye et al., 2012) and listed below.

CCK:

Forward 5′‐TCCCTGCAGAAGCTACGGAC‐3′
Backward 5′‐GGTCACCATCCATGCCACTTT‐3′

mGAPDH:

Forward 5′‐AGGTCGGTGTGAACGGATTTG‐3′
Backward 5′‐GGGGTCGTTGATGGCAACA‐3′

### 2.6 Immunofluorescence

Immunofluorescence was performed as previously described (Luo et al., 2022). Briefly, STC‐1 cells were fixed with 4% paraformaldehyde (PFA) (Cat. No. SW3015, Solarbio, Co. China) and permeabilized with 0.1% Triton X‐100 (Cat. No. ST1723‐100 mL, Beyotime Biotechnology, Co. China). The following primary antibodies were used: anti‐TRPV4 (Cat. No. ACC‐034, Alomone, Co. Israel, 1:100) or NCX1 (Cat. No. ANX‐011, Alomone, Co. Israel, 1:150). Meanwhile, the isotype control antibody (Cat. No. RIC‐001, Alomone, Co. Israel, 1:100) was applied for the control test. Incubation with primary antibodies was 1.5 h at +4°C and Alexa Fluor® 488 Conjugated Goat Anti‐Rabbit IgG H&L secondary antibodies (Cat. No. ab150077, Abcam, Co. United Kingdom, 1:500) for 1 h at room temperature, and then stained with 4,6‐Diamidino‐2‐Phenylindole (DAPI) (Cat. No. C1006, Beyotime Biotechnology, Co. China), at room temperature for 20 min. After finding a clear image under low magnification, a 40× oil objective lens was used for fine‐tuning to obtain a clear image under high magnification. Then, the DAPI and related channel antibodies were imaged and captured through the excitation light of channels 405 and 488 to obtain clear fluorescence images of each channel. Images were acquired with a confocal microscope (A1R MP, NIKON, Co. Japan), using 40× oil objective.

### 2.7 ELISA

ELISA kits of mouse CCK were obtained from Enzyme‐linked Biotechnology (Cat. No. ml058435, Shanghai Enzyme‐linked Biotechnology Co. China). Cell supernatant extraction and ELISA assays were performed according manufacturer's instructions. Briefly, cells were seeded onto 24‐well cell culture plates (Cat. No. YA0584, Solarbio, Co. China) at a density of 2 × 10^5^ per well. The following day, drugs were administered for a two‐hour treatment period, and then 200 μL supernatant was centrifuged at 1 × 10^3^ g for 10 min to eliminate particulate matter. 50 μL of the resulting solution was then added to 96‐well plate (Cat. No. YA0585, Solarbio, Co. China) precoated with CCK antibody. Sequential addition of various reagents followed the provided instructions. The optical density (OD) was measured at a wavelength of 450 nm for each well. The CCK concentration of each sample can be determined from these OD values utilizing the standard curve. The technique was repeated three times, with *n* = 3 for each group of samples.

### 2.8 Western blot analysis

Cells were lysed with Western and IP cell lysis buffer (Cat. No. P0013, Beyotime Biotechnology, Co. China) and centrifuged to obtain the supernatant. Protein content was determined using a BCA kit (Cat. No. P0010, Beyotime Biotechnology, Co. China). Total cell lysates were separated by SDS‐PAGE on 10% denaturing gel and then transferred to PVDF membrane (Cat. No. ISEQ00010, Millipore, Billerica, MA, USA). The blots were blocked with 5% skim milk at room temperature for 2 h and then incubated overnight at 4°C with the following specific primary antibodies: anti‐TRPV4 (Cat. No. ACC‐034, Alomone, Co. Israel, 1:100), anti‐NCX1 (Cat. No. ANX‐011, Alomone, Co. Israel, 1:150), and anti‐GAPDH (Cat. No. AG0191, Beyotime Biotechnology, Co. China, 1:1000). After washing, the blot was incubated with horseradish peroxidase (HRP)‐conjugated anti‐rabbit or anti‐mouse (Cat. No. A0239 and A0216, Beyotime Biotechnology, Co. China, 1:5000) secondary antibodies at room temperature for 1 h. Immunoreactive bands were detected using enhanced chemiluminescence (Cat. No. 34094, Thermo, Waltham, MA, Co. USA).

### 2.9 Co‐immunoprecipitation

Cells were lysed with immunoprecipitation lysis buffer for 30 min, then centrifuged at 12,000 g for 15 min to separate the supernatant. The supernatant was incubated overnight at 4°C with anti‐TRPV4 (Cat. No. ACC‐034, Alomone, Co. Israel, 1:100) and anti‐NCX1 (Cat. No. ANX‐011, Alomone, Co. Israel, 1:150) primary antibodies, followed by incubation with protein G agarose (Cat. No. C0130‐5 ml, Univ‐bio, Co. China) at room temperature for 2 h. After incubation, the protein G agarose was washed five times with ice‐cold PBS buffer and then boiled in 1 × SDS loading buffer for immunoblot analysis.

### 2.10 IBS‐D animal model establishment

The wrap restraint stress (WRS) was introduced in the scientific literature more than 30 years ago as the model for human IBS (Gué et al., 1997; Vannucchi & Evangelista, 2018; Williams et al., 1988; Wu et al., 2022). After 1 week of adaptive feeding, mice were divided into five groups randomly: control, model and 3 interventional groups. To minimize the influence of circadian rhythms, acute WRS was performed between 9 am and 11 am for 7 days. For mice in model group, i.p. injection of solvent was performed half an hour prior to stress induction every day. In contrast, for mice in interventional groups, i.p. injection of drugs was performed half an hour before stress every day. As for the control group, i.p. injection of solvent took place at 8:30 am every day. On the seventh day, fecal samples were collected from each mouse group and analyzed for weight measurement as well as moisture content determination along with quantification of distinguishable particles. At the end of the experiment, all experimental animals were killed by cervical dislocation under anesthesia with 100% CO_2_, and their colon and duodenum tissues were collected for qPCR experiments.

### 2.11 Data and statistical analysis

All quantitative, normally distributed data were analyzed for statistical significance using a Student's *t*‐test, One‐way ANOVA, with Tukey's post hoc tests when comparing more than two groups relative to a single factor. GraphPad Prism Software (GraphPad Prism version 8.0.2 for Windows; GraphPad Software, www.graphpad.com) was used.

### 2.12 Materials

Carbachol (CCh) (Cat. No. C‐4382, SIGMA, Co. USA). HC067047 (Cat. No. HY‐100208, MCE, Co. USA), SN 6 (Cat. No. HY‐107658, MCE, Co. USA), KB‐R7943 (Cat. No. HY‐15415, MCE, Co. USA), 2‐APB (2‐Aminoethyl Diphenylborinate) (Cat. No. HY‐W009724, MCE, Co. USA), Xestospongin C (Cat. No. HY‐103312, MCE, Co. USA), Devazepide (Cat. No. HY‐106301, MCE, Co. USA) were purchased from MedChemExpress. NaCl, KCl, CaCl_2_, MgCl_2_, HEPES, glucose, CsCl, EGTA, Mg‐ATP, CsOH, and NaOH were purchased from Aladdin Biochemical Technology in Shanghai, China. CCh was dissolved in pure water, and the remaining reagents are dissolved in DMSO (Cat. No. D8371, Solarbio, Co. China), the final concentration of DMSO was less than 0.1% and did not change the viability of experimental cells.

## 3 RESULTS

### 3.1 Carbachol elicited intracellular Ca^2+^ signaling and augmented non‐selective transmembrane currents in STC‐1 cells

It has been well documented that under physiological state enterohormone release from EECs is tightly controlled by the vagus nerve that releases neurotransmitter ACh to activate muscarinic receptors (MR) when stimulated (Zhang et al., 2026). Due to the instability of ACh, we utilized its stable analogue carbachol (CCh) in our experiments. Initially, we conducted patch‐clamp to examine the effect of CCh on the membrane currents of STC‐1 cells. Transmembrane currents were recorded at 100 mV and −100 mV. Following the addition of CCh (20 μM), a significant increase in membrane current was observed (Figure 1a), indicating activation of MR in EECs by CCh (20 μM). The current density‐voltage relationship curve demonstrates that the involved ion channels are non‐selective cation channels as the reversal potential was close to 0 mV (Figure 1b). Analysis based on multiple cell statistics further confirms a substantial enhancement in current densities caused by CCh (20 μM) (Figure 1c). Therefore, CCh stimulation of MR activates non‐selective cation channels in EECs.

**FIGURE 1:**
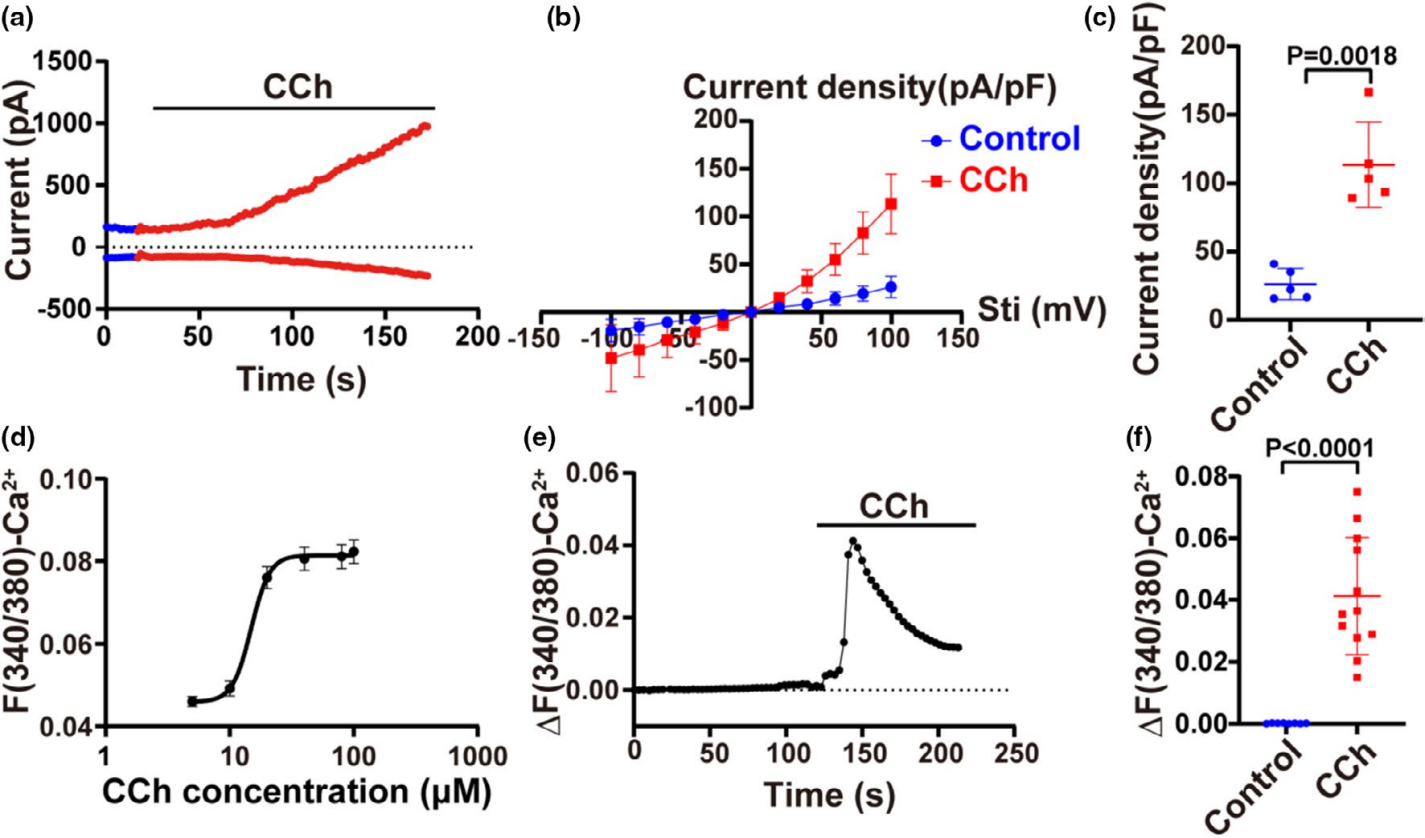
CCh enhanced transmembrane current densities and Ca^2+^ signaling in STC‐1. (a and b) Time course of the changes in the CCh‐induced current curves. (*n* = 5). (c) Summary data of current densities measured at 100 mV. (*n* = 5). (d) EC_50_ of CCh in STC‐1 is about 15 μM. (*n* = 3). (e) The time courses of CCh (20 μM)‐induced intracellular Ca^2+^ signaling in physiological salt solution (PSS) (*n* = 18 cells). (f) Comparisons of the Ca^2+^ signaling of control and CCh group. The sample number of CCh and control group is 16. Data are reported as mean −/+ standard deviation.

To validate if MR stimulation can induce Ca^2+^ entry via non‐selective cation channels in EECs, real‐time Ca^2+^ signaling was determined in single STC‐1 cells with fura‐2 calcium fluorescent dye. Moreover, the EC_50_ of CCh to increase Ca^2+^ in STC‐1 was measured to be 15 μM (Figure 1d). As shown in Figure 1e, CCh (20 μM) stimulation indeed induced significant Ca^2+^ signaling in STC‐1 cells. The observed substantial augmentation in intracellular Ca^2+^ signaling induced by CCh (20 μM) was statistically analyzed across multiple STC‐1 cells (Figure 1f). These findings indicate that CCh stimulation of MR induces Ca^2+^ signaling via non‐selective cation channels in EECs.

### 3.2 CCh enhanced Ca^2+^ signaling via IP_3_ receptor and Orai1 to activate non‐selective cation currents in STC‐1 cells

As a typical G protein‐coupled receptor, CCh stimulation of MR results in intracellular IP_3_ increase to induce endoplasmic reticulum (ER) Ca^2+^ release via IP_3_ receptors (IP_3_R), leading to regulation of non‐selective cation channels in EECs (Cui et al., 2021; Rosa et al., 2020). At the same time, CCh stimulation of MR also induces the release of Ca^2+^ from the ER through STIM1/Orai1 (Sabourin et al., 2015). We further examined these possibilities in STC‐1 cells. Initially, 2‐APB (100 μM), a commonly used IP_3_R blocker, significantly suppressed CCh (20 μM) enhanced transmembrane currents (Figure 2a). Furthermore, the current density‐voltage curves for the same cell highlight the CCh‐induced non‐selective cation current density following treatment with 2‐APB (100 μM) (Figure 2b). Statistical analysis using multiple cells confirms that 2‐APB significantly inhibited CCh enhanced current densities (Figure 2c). Secondly, pretreatment with 2‐APB (100 μM) for 30 min markedly attenuated CCh‐induced Ca^2+^ signaling (Figure 2d,e), which was further supported by statistical analysis showing significant inhibition of CCh‐induced Ca^2+^ signaling by 100 μM 2‐APB across multiple STC‐1 cells (Figure 2f). Then we further selected the IP_3_R selective blocker Xestospongin C (Xes 100 nM); it also significantly suppressed CCh (20 μM) enhanced transmembrane currents (Figure 2g), and the current density‐voltage curves for the same cell highlight the CCh (20 μM)‐induced non‐selective cation current density following treatment with Xes (100 nM) (Figure 2h). Statistical analysis using multiple cells confirms that Xes (100 nM) significantly inhibited CCh (20 μM) enhanced current densities (Figure 2i).

**FIGURE 2:**
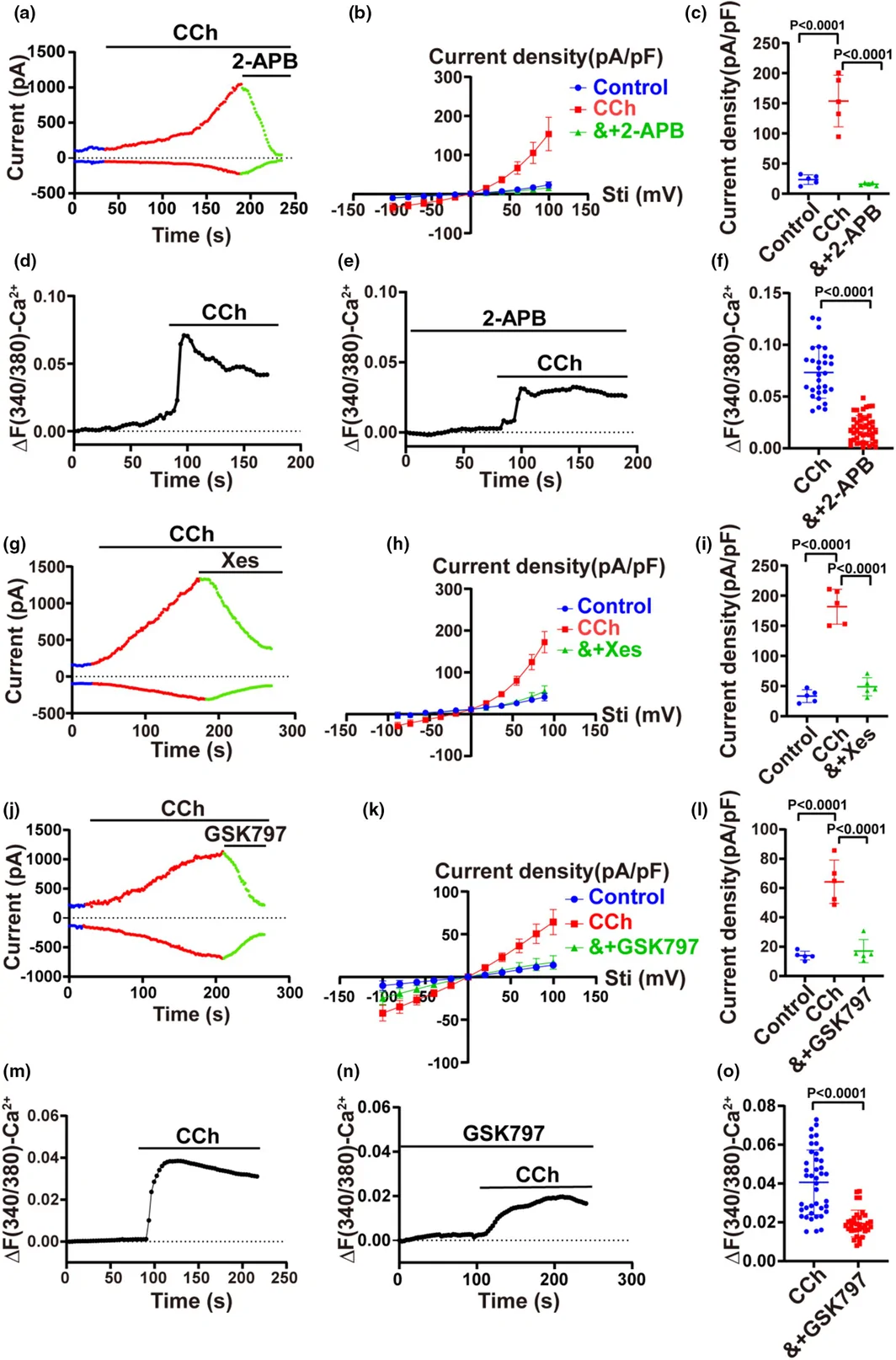
CCh‐induced increased Ca^2+^ signaling and transmembrane currents through IP_3_R pathway in STC‐1. (a and b) Time course of CCh (20 μM)‐induced changes in current curves with or without 2‐APB (100 μM). (*n* = 5). (c) Summary data of current densities measured at 100 mV. (*n* = 5). (d and e) Time course of changes in the CCh (20 μM)‐induced Fura‐2 ratio [Ca^2+^]_i_ with or without 2‐APB (100 μM) pretreatment. (f) Summary data of the delta Fura‐2 fluorescence ratio. (*n* = 32 cells and 45 cells). (g and h) Time course of CCh (20 μM)‐induced changes in current curves with or without Xestospongin C (Xes 100 nM). (*n* = 5). (i) Summary data of current densities measured at 100 mV. (*n* = 5). (j and k) Time course of CCh (20 μM)‐induced changes in current curves with or without GSK7975A (GSK797 10 μM). (*n* = 5). (l) Summary data of current densities measured at 100 mV. (*n* = 5). (m and n) Time course of changes in the CCh (20 μM)‐induced Fura‐2 ratio [Ca^2+^]_i_ with or without GSK7975A (GSK797 10 μM) pretreatment. (o) Summary data of the delta Fura‐2 fluorescence ratio (*n* = 41 cells and 34 cells). Data are reported as mean −/+ standard deviation.

Finally, commonly used Orai1 blocker GSK7975A (10 μM) significantly inhibited the transmembrane current enhanced by CCh (20 μM) (Figure 2j). Additionally, the current density‐voltage curve of the same cell after treatment with GSK7975A (10 μM) highlighted the CCh‐induced non‐selective cation current density (Figure 2k). Statistical analysis of multiple cells confirmed that GSK7975A significantly inhibited the CCh‐enhanced current density (Figure 2l). Then, 30‐min pretreatment with GSK7975A (10 μM) significantly weakened the CCh‐induced Ca^2+^ signaling (Figure 2m,n). Further statistical analysis indicated that 10 μM GSK7975A significantly inhibited the CCh‐induced Ca^2+^ signaling in multiple STC‐1 cells (Figure 2o). Taken together, these data strongly suggest that MR stimulation causes ER/Ca^2+^ release via IP_3_R and Orai1 to enhance Ca^2+^ entry via non‐selective cation channels in EECs.

### 3.3 Expression and function of TRPV4 channels in STC‐1 cells

We further examined which specific non‐selective cation channels are involved in MR/IP_3_R/Ca^2+^ and MR/STIM1/Orai1/Ca^2+^‐mediated processes in EECs. We focused on TRPV4 channels since, as nonselective cation channels with high Ca^2+^ permeability, they can be activated by IP_3_R/Ca^2+^ signaling and are closely related to IBS (Cui et al., 2021). However, the expression and function of TRPV4 channels have not been reported previously in STC‐1 cells. To test the functionality of TRPV4 channels in STC‐1 cells, first, a selective activator of TRPV4 channels, RN‐1747 (20 μM), markedly enhanced membrane non‐selective cation currents, which was abolished by a selective blocker of TRPV4 channels, HC067047 (10 μM) (Figure 3a). The current density‐voltage curves for the same cell highlight the non‐selective cation current density induced by RN‐1747 (20 μM) following treatment with HC067047 (10 μM) (Figure 3b). After examining multiple cells, it was observed that HC067047 (10 μM) had a significant inhibitory effect on RN‐1747 (20 μM)‐induced current densities (Figure 3c).

**FIGURE 3:**
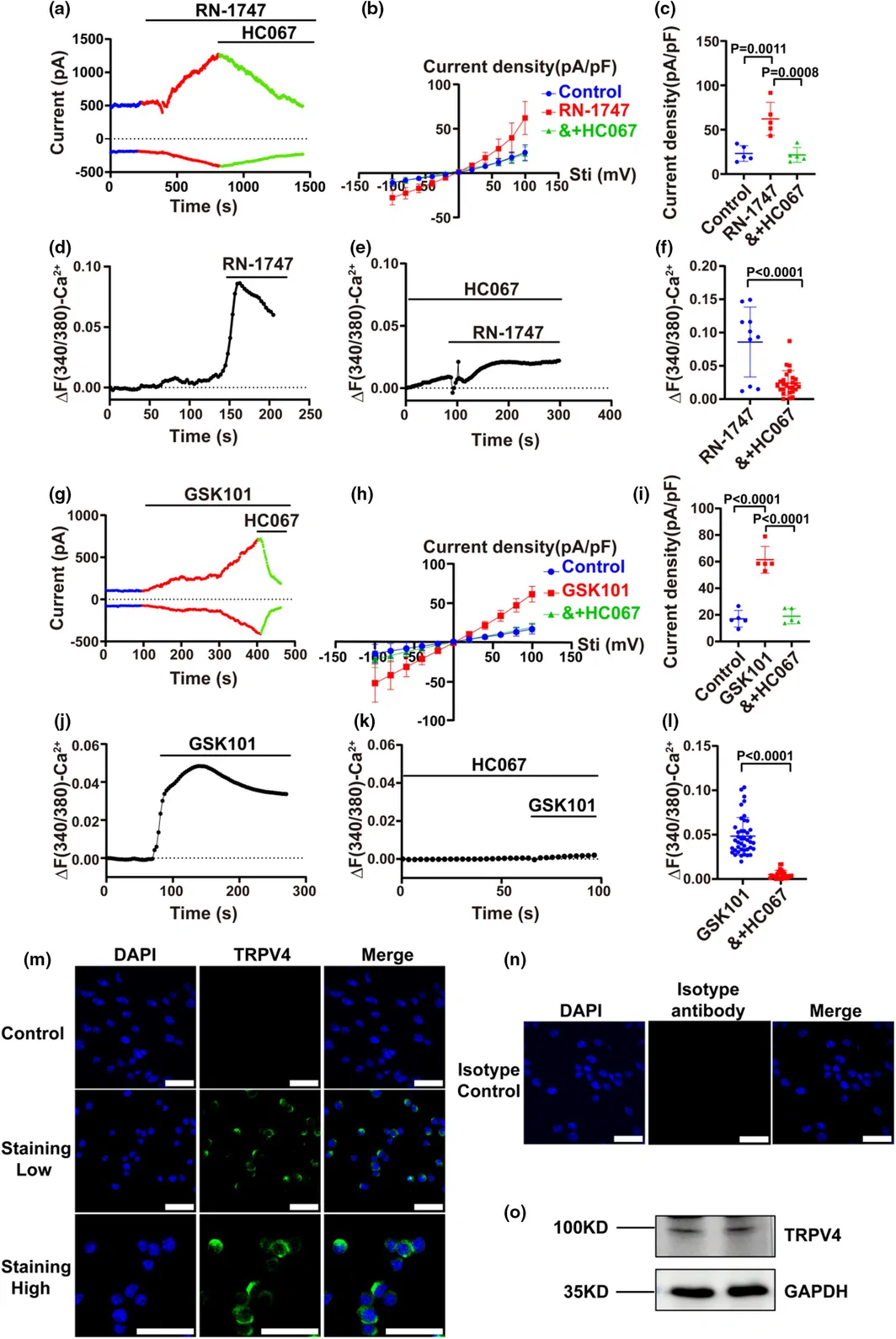
Expression and function of TRPV4 channels in STC‐1. (a and b) Time course of RN‐1747 (20 μM)‐induced changes in current curves with or without HC067047 (HC067 10 μM) (*n* = 5). (c) Summary data of current densities measured at 100 mV (*n* = 5). (d and e) Time course of changes in the RN‐1747 (20 μM)‐induced Fura‐2 ratio [Ca^2+^]_i_ with or without HC067047(HC067 10 μM) pretreatment. (f) Summary data of the delta Fura‐2 fluorescence ratio (*n* = 10 cells and 26 cells). (g and h) Time course of GSK101 (100 nM)‐induced changes in current curves with or without HC067047 (HC067 10 μM) (*n* = 5). (i) Summary data of current densities measured at 100 mV (*n* = 5). (j and k) Time course of changes in the GSK101 (100 nM)‐induced Fura‐2 ratio [Ca^2+^]_i_ with or without HC067047(HC067 10 μM) pretreatment. (l) Comparisons of the Ca^2+^ signaling of GSK101 (100 nM) and GSK101 + HC067047 (HC067 10 μM).(*n* = 42 cells and 26 cells). (m) Immunofluorescence staining of TRPV4 channels in STC‐1 s. The first line: No primary antibody against TRPV4 as a negative control. The scale bar, depicted in white, measures 50 μm. The second line: The lower staining to TRPV4 protein (in green), the nuclei of the cells stained with DAPI (in blue). The white scale bar is 50 μm.(40× lens) The third line: The higher staining to TRPV4 protein (in green), the nuclei of the cells stained with DAPI (in blue). The scale bar, depicted in white, measures 50 μm.(Select the local image in the second line and enlarge it by 4×) (n) Isotype antibody staining, nuclear staining is DAPI (blue). The white scale is 50 μm (*n* = 3). (o) Western blot confirmed the expression of TRPV4 protein in STC‐1 cells (*n* = 3). Each lane represents two independent experiments. Data are reported as mean −/+ standard deviation.

Second, RN‐1747 (20 μM) enhanced a marked Ca^2+^ signaling in STC‐1 cells (Figure 3d), which was abolished by pre‐treatment with HC067047 (10 μM) for 30 min (Figure 3e). Multiple cell counts further confirmed the significant inhibition of HC067047 (10 μM) on RN‐1747 (20 μM)‐induced Ca^2+^ signaling (Figure 3f).

Meanwhile, another selective TRPV4 channel agonist, GSK1016790A (GSK101, 100 nM), significantly enhanced the membrane non‐selective cation current, which was abolished by a selective blocker of TRPV4 channels HC067047 (10 μM) (Figure 3g). The current density‐voltage curves for the same cell highlight the non‐selective cation current density induced by GSK101 (100 nM) following treatment with HC067047 (10 μM) (Figure 3h). After examining multiple cells, it was observed that HC067047 (10 μM) had a significant inhibitory effect on GSK101 (100 nM)‐induced current densities (Figure 3i).

Then, GSK101 (100 nM) enhanced marked Ca^2+^ signaling in STC‐1 cells (Figure 3j), which was abolished by pre‐treatment with HC067047 (10 μM) for 30 min (Figure 3k). Multiple cell counts further confirmed the significant inhibition of HC067047 (10 μM) on GSK101 (100 nM)‐induced Ca^2+^ signaling (Figure 3l).

Immunofluorescence analysis showed protein expression of TRPV4 in STC‐1; however, control without primary anti‐TRPV4 antibody and the control group with the isotype antibody did not exhibit any green fluorescence (Figure 3m,n), indicating specific staining of TRPV4 proteins. We also detected the protein expression of TRPV4 channels on STC‐1 cells by Western blot (Figure 3o).

Finally, taken together, these results provide strong evidence for the expression and function of TRPV4 channels in STC‐1 cells.

### 3.4 TRPV4 channels are involved in cholinergic signaling in STC‐1 cells

To further determine if TRPV4 channels are involved in cholinergic signaling in STC‐1 cells, selective blockers of TRPV4 channels were applied. As shown in Figure 4a, CCh (20 μM)‐induced membrane currents were abolished by HC067047 (10 μM). Current density‐voltage curves for the same cell highlight the non‐selective cation current density by CCh (20 μM) following treatment with HC067047 (10 μM) (Figure 4b). Statistical analysis on multiple cells showed significant inhibition of current densities induced by CCh was significantly inhibited by HC067047 (10 μM) (Figure 4c). We also revealed that HC067047 (10 μM) significantly inhibited CCh (20 μM)‐induced Ca^2+^ signaling in STC‐1 cells (Figure 4d,e) and summary data were shown in Figure 4f. These results strongly suggest that TRPV4 channels are physiologically involved in cholinergic signaling in STC‐1 cells.

**FIGURE 4:**
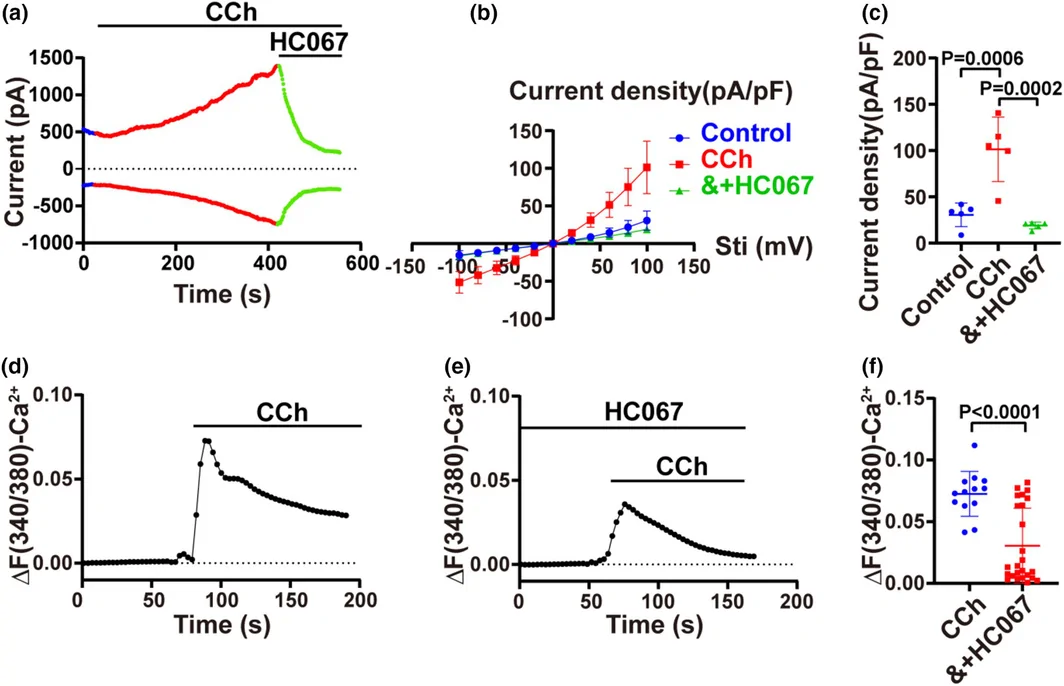
CCh increased Ca^2+^ signaling and transmembrane currents through TRPV4 channels. (a) CCh (20 μM) enhanced transmembrane currents and inhibited by HC067047 (10 μM) (*n* = 5). (b) Current density‐voltage curves in response to voltage steps from −100 to +100 mV in the presence of CCh (20 μM) or a combination of CCh plus HC067047 (HC067 10 μM) (*n* = 5). (c) Summary data of current densities measured at 100 mV (*n* = 5). (d, e) Time course of changes in the CCh (20 μM)‐induced Fura‐2 ratio [Ca^2+^]_i_ with or without HC067047 (HC067 10 μM) pretreatment. (f) Summary data of the delta Fura‐2 fluorescence ratio.(*n* = 13 cells and 25 cells). Data are reported as mean −/+ standard deviation.

### 3.5 Functionality of NCX1 in STC‐1 cells

Since Na^+^/Ca^2+^‐exchanger1 (NCX1) also plays a significant role in intracellular Ca^2+^ homeostasis in EECs but research on this topic remains limited, we proceeded with further investigation into the function of NCX1. Firstly, NCX1 operates in two modes: the forward mode facilitates the influx of Na^+^ into the cell and the efflux of Ca^2+^ from the cell, whereas the reverse mode results in the efflux of Na^+^ from the cell and the influx of Ca^2+^ into the cell. Application of CaCl_2_ could stimulate the reverse mode of NCX1. Indeed, CaCl_2_ (5 mM) resulted in a significant augmentation of the membrane currents, which were abolished by selective inhibitors for the reverse mode of NCX1, KB‐R7943 (30 μM) and SN6 (10 μM) (Figure 5a,d). The current density‐voltage curves analysis revealed that treatment with KB‐R7943 (30 μM) and SN 6 (10 μM) reduces the CaCl_2_ (5 mM)‐induced non‐selective cation current density (Figure 5b,e). The statistical analysis of multiple cells demonstrates significant inhibitory effects of KB‐R7943 (30 μM) and SN6 (10 μM) on CaCl_2_ (5 mM)‐induced current densities (Figure 5c,f). Immunofluorescence analysis showed protein expression of NCX1 in STC‐1; however, control without primary anti‐NCX1 antibody did not exhibit any green fluorescence (Figure 5g), and the protein expression of NCX1 on STC‐1 cells was also detected by Western blot (Figure 5h), indicating specific staining of NCX1 proteins. Therefore, NCX1 can operate in the reverse mode in EECs.

**FIGURE 5:**
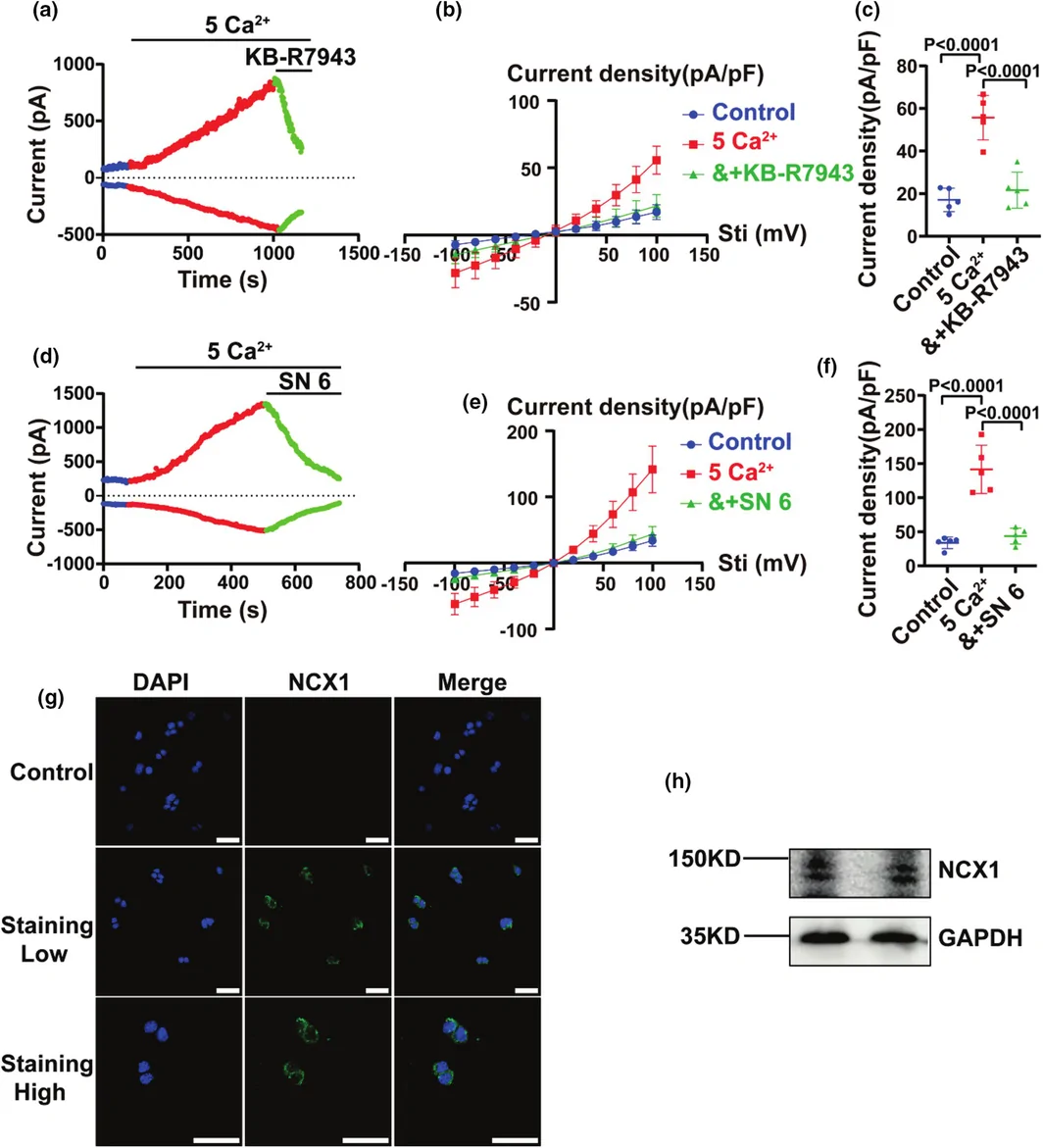
NCX1 operates in reverse mode in STC‐1 cells. (a and b) Time course of CaCl_2_ (5 Ca^2+^ 5 mM)‐induced changes in current curves with or without KB‐R7943 (30 μM) (*n* = 5). (c) Summary data of current densities measured at 100 mV (*n* = 5). (d and e) Time course of CaCl_2_ (5 Ca^2+^ 5 mM)‐induced changes in current curves with or without SN 6 (10 μM) (*n* = 5). (f) Summary data of current densities measured at 100 mV (*n* = 5). (g) Immunofluorescence staining of NCX1 in STC‐1s. The upper panel: No primary antibody against NCX1 as a negative control. The scale bar, depicted in white, measures 50 μm. The middle panels: The lower staining to NCX1 protein (in green), the nuclei of the cells stained with DAPI (in blue). The scale bar, depicted in white, measures 50 μm (40× lens). The lower panels: The higher staining to NCX1 protein (in green), the nuclei of the cells stained with DAPI (in blue). The white scale bar is 50 μm (*n* = 3). (Select the local image in the middle line and enlarge it by 4×) (h) Western blot confirmed the expression of NCX1 protein in STC‐1 (*n* = 3). Each lane represents two independent experiments. Data are reported as mean −/+ standard deviation.

### 3.6 The reverse mode of NCX1 is involved in cholinergic signaling in STC‐1 cells

To test whether the reverse mode of NCX1 is involved in cholinergic signaling in EECs, we initially applied CCh (20 μM) to STC‐1 cells, which induced membrane currents in STC‐1 cells; however, KB‐R7943 (30 μM) abolished CCh‐induced membrane currents (Figure 6a). The current density‐voltage curve for the same cell highlighting the enhanced current densities was mediated by non‐selective cation channels and reversed by KB‐R7943 (30 μM) (Figure 6b). The statistical analysis of multiple cells demonstrates a significant inhibitory effect of KB‐R7943 (30 μM) on CCh enhanced current densities (Figure 6c). Finally, we also applied SN 6 (10 μM), another well‐known antagonist of the reverse mode of NCX1. Upon stimulation with CCh (20 μM), STC‐1 displayed an elevation in its membrane currents, and subsequent addition of SN 6 (10 μM) abolished CCh‐induced membrane currents (Figure 6d). The current density‐voltage curve for the same cell highlighting the enhanced current densities was mediated by non‐selective cation channels and reversed by SN 6 (Figure 6e). By analyzing multiple cells, SN 6 (10 μM) exerts a pronounced inhibitory effect on CCh (20 μM) enhanced current densities (Figure 6f). Taken together, these data suggest the reverse mode of NCX1 is involved in cholinergic signaling in EECs.

**FIGURE 6:**
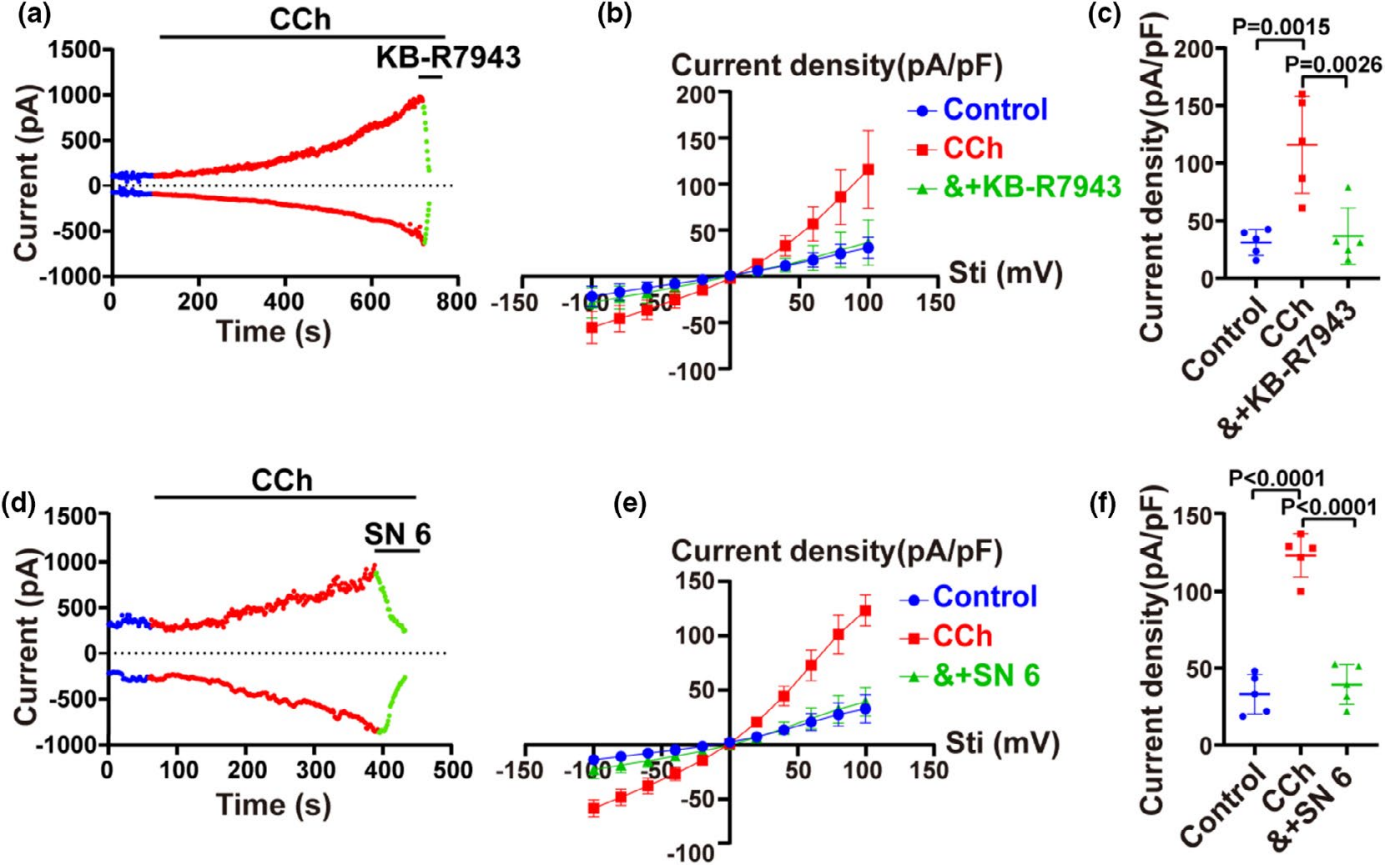
CCh enhanced transmembrane currents change through NCX1 transporter. (a) CCh (20 μM) enhanced transmembrane currents and can be significantly inhibited by KB‐R7943 (30 μM) (*n* = 5). (b) Current density‐voltage curves in response to voltage steps from −100 to +100 mV in the presence of CCh (20 μM) or a combination of CCh plus KB‐R7943 (30 μM) (*n* = 5). (c) Summary data of current densities measured at 100 mV (*n* = 5). (d) CCh (20 μM) enhanced membrane currents and can be significantly inhibited by 10 μM SN 6 (*n* = 5). (e) Current density‐voltage curves in response to voltage steps from −100 to +100 mV in the presence of CCh (20 μM) or a combination of CCh plus SN 6 (10 μM) (*n* = 5). (f) Summary data of current densities measured at 100 mV (*n* = 5). Data are reported as mean −/+ standard deviation.

We applied single cell Ca^2+^ imaging to further test the involvement of the reverse mode of NCX1 in cholinergic signaling in EECs. Pre‐treatment of STC‐1 cells with KB‐R7943 (30 μM) significantly inhibited CCh‐induced Ca^2+^ signaling (Figure 7a–c). Furthermore, pre‐treatment with SN 6 (10 μM) also significantly inhibited CCh‐induced Ca^2+^ signaling in STC‐1 cells (Figure 7d–f). These Ca^2+^ imaging data combined with patch‐clamp data strongly support our notion that the reverse mode of NCX1 is clearly involved in cholinergic signaling in EECs.

**FIGURE 7:**
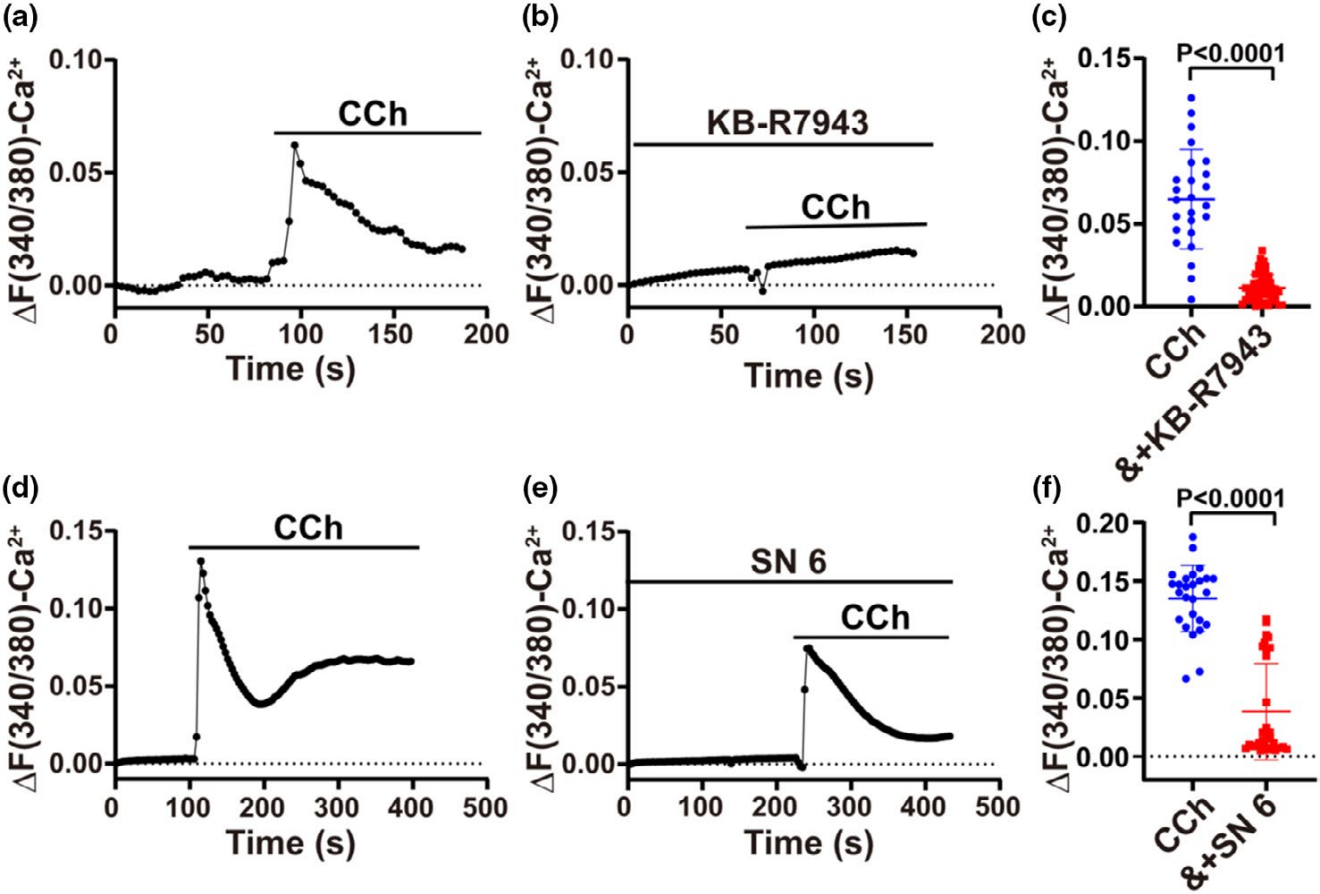
CCh‐induced Ca^2+^ signaling changes through NCX1 transporter. (a and b) Time course of changes in the CCh (20 μM)‐induced Fura‐2 ratio [Ca^2+^]_i_ with or without KB‐R7943 (30 μM) pretreatment. (c) Summary data of the delta Fura‐2 fluorescence ratio. (*n* = 21 cells and 46 cells). (d and e) Time course of changes in the CCh (20 μM)‐induced Fura‐2 ratio [Ca^2+^]_i_ with or without SN 6 (10 μM) pretreatment. (f) Summary data of the delta Fura‐2 fluorescence ratio (*n* = 23 cells and 29 cells). Data are reported as mean −/+ standard deviation.

### 3.7 TRPV4 and NCX1 functional coupling in STC‐1 cells

Since previous studies demonstrated TRPV4 channels and NCX1 coupling in tracheal smooth muscle (Zhang et al., 2019), we wondered if this coupling also exists in EECs. Therefore, selective activator of TRPV4 channels, RN‐1747, was first applied to STC‐1 cells then using a selective inhibitor of NCX1, SN 6. RN‐1747 (20 μM) significantly increased membrane currents, which were abolished by SN 6 (10 μM) (Figure 8a). Furthermore, the current density‐voltage curve for the same cell highlighted the non‐selective cation current density by RN‐1747 (20 μM) following treatment with SN 6 (10 μM) (Figure 8b). Statistical analysis across multiple cells confirmed that SN 6 (10 μM) has a significant inhibitory effect on RN‐1747 enhanced current densities (Figure 8c). Additionally, pre‐treatment with SN 6 (10 μM) reduced RN‐1747 (20 μM)‐induced Ca^2+^ signaling in STC‐1 cells (Figure 8d,e). The inhibitory effect of SN 6 (10 μM) on RN‐1747 (20 μM)‐induced Ca^2+^ signaling was demonstrated through the quantification of multiple cells (Figure 8f), highlighting its significance.

**FIGURE 8:**
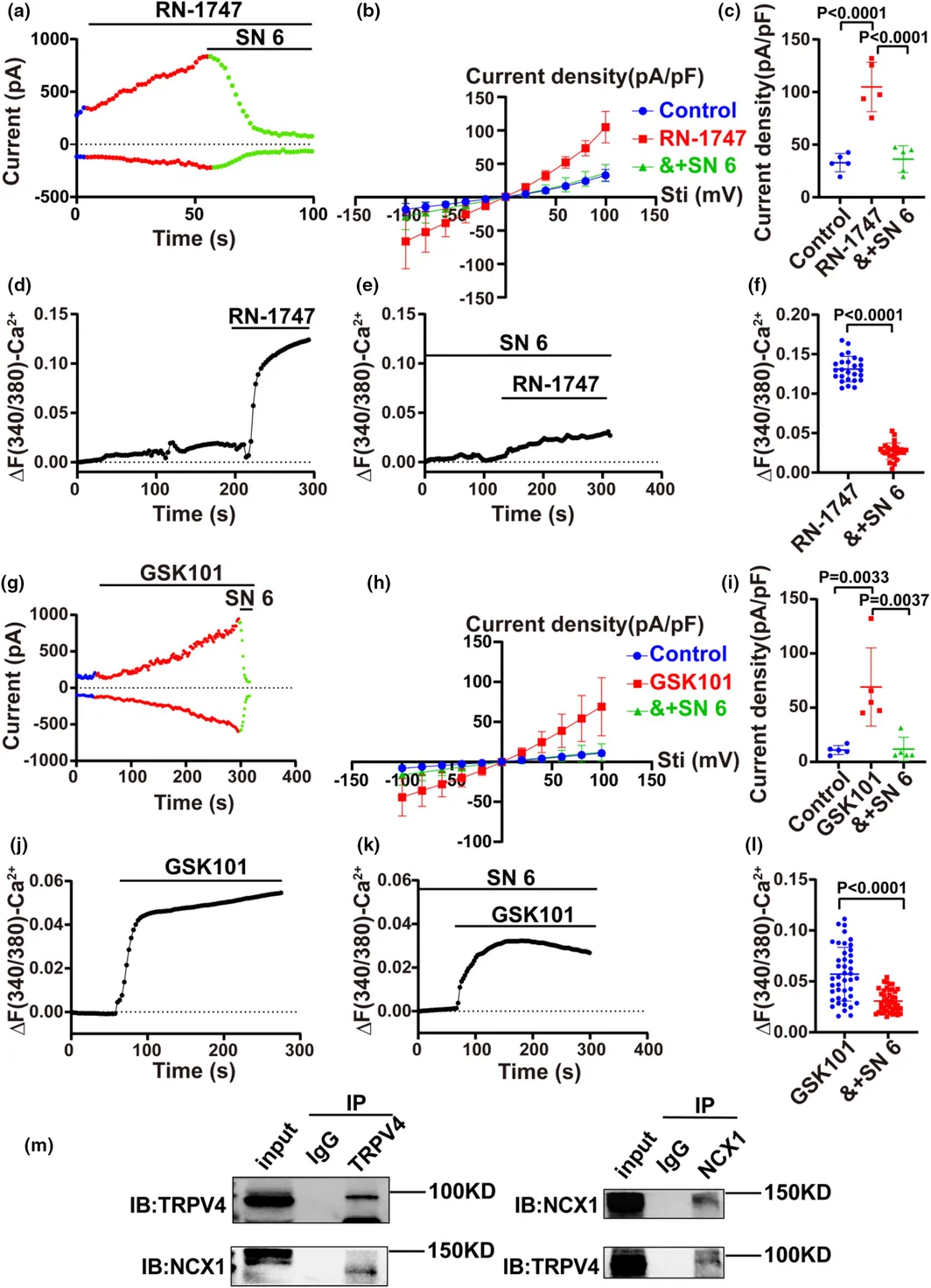
A functional coupling relationship exists between the TRPV4 channels and NCX1 in STC‐1 cells. (a and b) Time course of RN‐1747 (20 μM)‐induced changes in current curves with or without SN 6 (10 μM) (*n* = 5). (c) Summary data of current densities measured at 100 mV (*n* = 5). (d and e) Time course of changes in the RN‐1747 (20 μM)‐induced Fura‐2 ratio [Ca^2+^]_i_ with or without SN 6 (10 μM) pretreatment. (f) Summary data of the delta Fura‐2 fluorescence ratio (*n* = 23 cells and 26 cells). (g and h) Time course of GSK101 (100 nM)‐induced changes in current curves with or without SN 6 (10 μM) (*n* = 5). (i) Summary data of current densities measured at 100 mV (*n* = 5). (j and k) Time course of changes in the GSK101 (100 nM)‐induced Fura‐2 ratio [Ca^2+^]_i_ with or without SN 6 (10 μM) pretreatment. (l) Summary data of the delta Fura‐2 fluorescence ratio. (*n* = 43 cells and 43 cells). (m) Co‐immunoprecipitation showing the physical interaction of TRPV4 and NCX1 proteins in STC‐1 cells (*n* = 3). Data are reported as mean −/+ standard deviation.

We continued to use another TRPV4 channels selective agonist, GSK101. GSK101 was first applied to STC‐1 cells, and then the selective inhibitor of NCX1, SN 6, was used to further confirm the relationship between TRPV4 channels and NCX1. GSK101 (100 nM) significantly increased membrane currents, which were abolished by SN 6 (10 μM) (Figure 8g). Furthermore, the current density‐voltage curve for the same cell highlighted the non‐selective cation current density by GSK101 (100 nM) following treatment with SN 6 (10 μM) (Figure 8h). Statistical analysis across multiple cells confirmed that SN 6 (10 μM) has a significant inhibitory effect on GSK101 enhanced current densities (Figure 8i). Additionally, pre‐treatment with SN 6 (10 μM) reduced GSK101 (100 nM)‐induced Ca^2+^ signaling in STC‐1 cells (Figure 8j,k). The inhibitory effect of SN 6 (10 μM) on GSK101 (100 nM)‐induced Ca^2+^ signaling was demonstrated through the quantification of multiple cells (Figure 8l), highlighting its significance. Then, we conducted co‐immunoprecipitation, which unequivocally demonstrated the physical interaction between TRPV4 and NCX1 proteins in STC‐1 cells (Figure 8m), thereby confirming their direct physical coupling.

These findings strongly suggest a functional coupling between TRPV4 and NCX1 in EECs.

### 3.8 The role of TRPV4/NCX1 coupling in the pathogenesis of IBS

Since enterohormone secretion from EECs is implicated in the pathogenesis of IBS (McCarthy et al., 2015; Qin et al., 2020). We wondered if TRPV4/NCX1 coupling that may regulate Ca^2+^‐dependent enterohormone secretion from EECs is possibly involved in IBS. First, a mouse model of IBS‐D was established by wrap restraint stress (WRS). Our results revealed significant increase in transcription levels of duodenum and colon CCK in WRS mice compared to their corresponding control mice (Figure 9a,b). Importantly, pre‐treatment with TRPV4 channels blocker HC067047 (10 mg.kg^−1^), NCX1 blocker KB‐R7943 (20 mg.kg^−1^), and CCK receptor antagonist devazepide (1 mg.kg^−1^) all significantly suppressed the increase in transcription levels of duodenum and colon CCK in WRS mice (Figure 9a,b). Secondly, we directly determined CCK secretion using ELISA kit. CCh (20 μM) significantly elevated CCK secretion compared to the control group. Notably, the CCh‐induced CCK secretion was markedly suppressed by HC067047 (10 μM), KB‐R7943 (30 μM), and SN 6 (10 μM) (Figure 9c).

**FIGURE 9:**
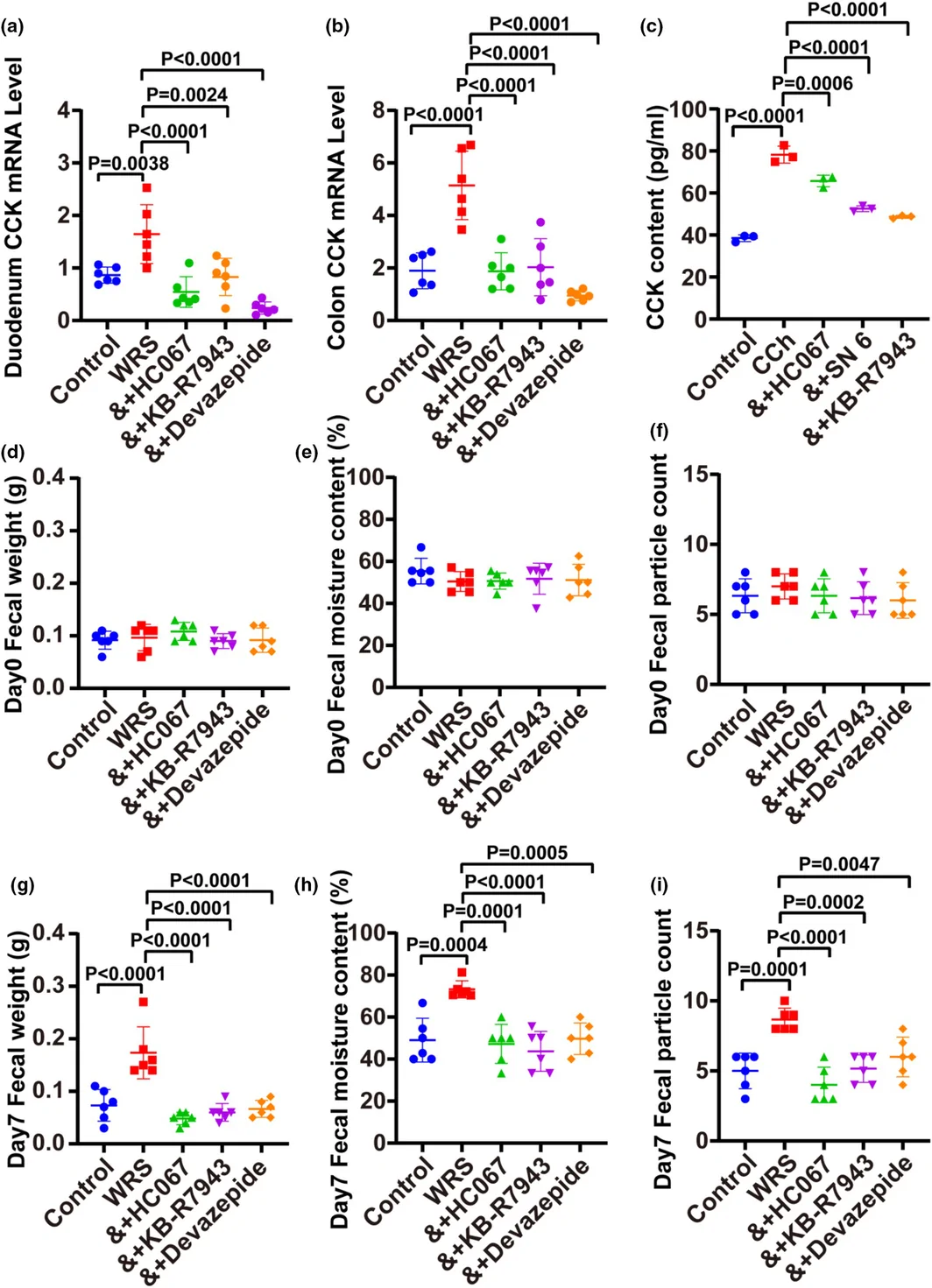
Involvements of TRPV4, NCX1, and CCK in the pathogenesis of IBS‐D. (a and b) Summary data on the transcription of CCK in duodenum and colon in control, WRS and apply HC067047 (10 mg.kg^−1^), KB‐R7943 (30 mg.kg^−1^), Devazepide (1 mg.kg^−1^) groups (*n* = 6). (c) Summary data on the content of CCK in cell culture supernatants following treatment with CCh (20 μM), CCh + HC067047 (10 μM), CCh + KB‐R7943 (30 μM), and CCh + SN 6 (10 μM) (*n* = 3). (d–f) Summary data showing the fecal weight and dry wet ratio of feces of control, WRS and apply HC067047 (10 mg.kg^−1^), KB‐R7943 (20 mg.kg^−1^), Devazepide (1 mg.kg^−1^) group at the day 0 (*n* = 6). (g–i) Summary data showing the fecal weight, water content and number of fecal particles of control, WRS and apply HC067047 (10 mg.kg^−1^), KB‐R7943 (30 mg.kg^−1^), Devazepide (1 mg.kg^−1^) groups (*n* = 6). Data are reported as mean ± standard deviation.

Third, before being restrained, the number, weight, and water content of feces were measured among each group of mice; there was no significant difference between each group (Figure 9d–f). However, after being restrained, the number, weight, and water content of feces in the WRS group mice were significantly increased compared to the control group (Figure 9g–i), indicative of the signs of diarrhea associated with IBS‐D. Forth, administration of 10 mg.kg^−1^ HC067047, 20 mg.kg^−1^ KB‐R7943, and 1 mg.kg^−1^ devazepide significantly restored fecal weight, water content, and number of fecal particles in restrained mice (Figure 9g–i). Taken together, these findings obtained from in vivo study strongly suggest that TRPV4/NCX1 coupling plays a pivotal role in Ca^2+^‐dependent CCK secretion, which is likely involved in the pathogenesis of IBS‐D.

## 4 DISCUSSION

The effective prevention and therapy for IBS have been currently hindered due to its intricate etiology and our incomplete understanding of its pathogenesis. Although enterohormone CCK is known to be involved in IBS (Furgała et al., 2023), it is still obscure for the regulatory mechanisms of CCK secretion from EECs and the molecular pathogenesis of CCK‐associated IBS. In the present study, our principal findings are as follows: (1) vagus nerve neurotransmitter could activate muscarinic receptors (MR) in enteroendocrine cells (EECs), leading to an increase in membrane currents and intracellular Ca^2+^ signaling (Figure 1); (2) TRPV4/NCX1 coupling plays a crucial role in cholinergic signaling to regulate Ca^2+^‐dependent CCK production and secretion in EECs (Figure 8); (3) the duodenum and colon from IBS‐D mice exhibited greater CCK transcription, which could be abolished by selective blockers of TRPV4 channels, NCX1, and CCK receptors (Figure 9a,b); (4) intraperitoneal administration of the selective blockers of TRPV4 channels, NCX1, and CCK receptors effectively restored the increased fecal weight, water content, and number of fecal particles in IBS‐D mice (Figure 9g–i). Therefore, stress may enhance cholinergic signaling to upregulate Ca^2+^‐dependent CCK secretion via TRPV4/NCX1 coupling in EECs, leading to IBS‐D (as shown in Figure 10). Therefore, we reveal a molecular mechanism in the brain–gut axis via cholinergic signaling in EECs, which is also involved in the pathogenesis of IBS. Furthermore, TRPV4 channels and NCX1 could serve as potential targets for the prevention/treatment of IBS‐D.

**FIGURE 10:**
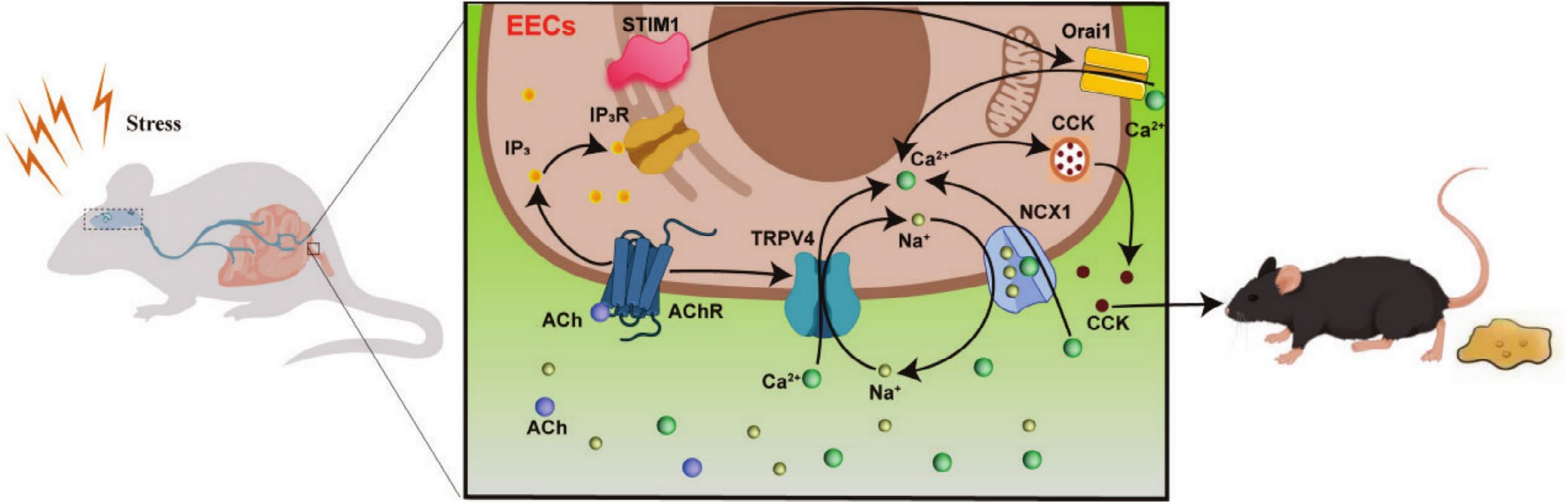
Schematic of the proposed TRPV4/NCX1/Ca^2+^/CCK pathway in stress‐induced IBS‐D. Under stress, the vagus nerve is stimulated and releases the neurotransmitter ACh, which binds to ACh receptors (Figure 1), triggering IP_3_‐mediated activation of IP_3_R and simultaneously activating Orai1, which leads to an enhanced intracellular Ca^2+^ signaling (Figure 2), subsequently resulting in opening of the TRPV4 channel on the membrane (Figures 3 and 4). TRPV4 collaborates with NCX1 to enhance Ca^2+^ signaling (Figures 5, 6, 7, 8), thereby facilitating the release of CCK and ultimately triggering IBS‐D (Figure 9).

The intestinal epithelium has evolved specialized EECs that exhibit conserved sensory functions and enterohormone secretions in mammals. Distributed along the entire digestive tract, EECs are activated by diverse luminal stimuli to secrete hormones or neuronal transmitters in a Ca^2+^‐dependent manner (Kumar et al., 2021; Petersen et al., 2023). The vertebrate intestine is innervated by the intrinsic enteric nervous system (ENS) and extrinsic neurons from vagal ganglia to connect to the central nervous system (CNS). Although the CNS and EECs regulate gastrointestinal and brain functions through the connection of the brain–gut axis (Barakat et al., 2024), however, the molecular mechanisms involved are largely unknown. Stress‐induced vagus nerve neurotransmitter could activate MR in EECs, leading to Ca^2+^ entry via ion channels and transporters. However, little is currently known about the expression and function of specific ion channels and transporters that contribute to the enterohormone secretion in EECs. We reveal for the first time that cholinergic signaling mediates intracellular Ca^2+^ in STC‐1 cells by stimulating ER/Ca^2+^ release via IP_3_R, leading to activation of TRPV4 channels since: (1) the expression of TRPV4 channels was shown in STC‐1 cells by immunofluorescence analysis and western blot (Figure 3m–o); (2) the function of TRPV4 channels was verified in STC‐1 cells by patch‐clamp and Ca^2+^ imaging (Figures 3 and 4); (3) CCh‐induced Ca^2+^ signaling and membrane currents could be abolished by selective blockers of IP_3_R and TRPV4 channels (Figures 2 and 4). Afterwards, we verified that NCX1 could operate in reverse mode in STC‐1 cells by using patch‐clamp and Ca^2+^ imaging and that the reverse mode of NCX1 is also involved in cholinergic signaling in EECs (Figures 5, 6, 7).

Previous studies showed a functional coupling between TRPV4 channels and NCX1 in tracheal smooth muscle (Zhang et al., 2019). Moreover, numerous studies demonstrated the functionality of either TRPV4 channels or NCX1 in intestinal epithelial cells (Cantero‐Recasens et al., 2019; Cui et al., 2021; Nishiyama et al., 2016; Zheng et al., 2020); however, it is unclear whether TRPV4 channels and NCX1 couple to regulate Ca^2+^‐dependent enterohormone secretion. Indeed, we have provided the following evidence for the existence of this coupling in EECs: (1) the physical interaction of TRPV4 and NCX1 proteins was confirmed by patch‐clamp and Co‐immunoprecipitation experiment in STC‐1 cells (Figure 8); (2) in patch‐clamp experiment, TRPV4 channel selective agonist RN‐1747‐induced membrane currents in STC‐1 cells were abolished by SN 6, a selective inhibitor of reverse mode NCX1 (Figure 8); (3) in Ca^2+^ imaging study, RN‐1747‐induced Ca^2+^ signaling was abolished by SN 6 (Figure 8), further supporting this coupling in EECs. These data further support our previous notion that TRPV4/NCX1 coupling is involved in glucose‐induced GLP‐1 secretion from EECs (Chen et al., 2024).

TRP channels have been shown to closely related to the pathogenesis or progression of IBS‐D (Cheng et al., 2022; Choi et al., 2024). Among TRP channel family, TRPV4 is mostly associated with IBS‐D in animals and humans, and even the increased levels of endogenous TRPV4 agonists were observed in IBS‐D patients (Beckers et al., 2017). Although the pathogenesis role of CCK in IBS has been demonstrated (Furgała et al., 2023), it remains unclear about the upstream molecular mechanisms of CCK secretion mediated by the vagus nerve via cholinergic signaling in EECs. Furthermore, since TRPV4/NCX1 coupling regulates Ca^2+^‐dependent CCK secretion from EECs, we wonder if this process contributes to the development of IBS‐D. Indeed, we demonstrate that intestine obtained from IBS‐D mice exhibited greater CCK transcription and secretion, which could be significantly abolished by the selective blockers of TRPV4 channels, NCX1 and CCK receptors. These data strongly suggest that TRPV4/NCX1 coupling via regulation of Ca^2+^‐dependent CCK secretion from EECs contributes to the development of IBS‐D, highlighting the pivotal role of ion channels and transporters in cholinergic signaling of EECs in IBS.

By using an acknowledged IBS‐D mouse model of wrap restraint stress (WRS), it is scientifically acknowledged that “female mice undergo periodic fluctuations in their emotional states and sensitivity” (Marchette et al., 2018), so since the hormone cycle of female mice may interfere with the stability of the IBS‐D model, we did not include female mice in the study. We showed that IBS mice exhibited a marked increase in defecation volume and symptoms of diarrhea, consistently with the symptoms of IBS‐D. We also observed a significant upregulation of CCK transcripts in the intestine of WRS mice, which could be abolished by selective blockers of TRPV4 channels, NCX1 and CCK receptors (Figure 9a,b), and why would CCK receptor blockers regulate the expression of the gene encoding CCK? It is not unexpected that receptor blockers may regulate the expression of ligand‐encoding genes by disrupting receptor‐mediated signaling networks, influencing transcription factors, feedback loops, or epigenetic states, ultimately modulating ligand gene expression. Such as in opioid receptor blockers (e.g., naloxone): long‐term use may upregulate the gene expression of endogenous opioid peptides (e.g., β‐endorphin) through negative feedback mechanisms. Also in angiotensin II receptor blockers (e.g., losartan): by blocking the AT1 receptor, elevated angiotensin II levels may activate the AT2 receptor, indirectly modulating renin gene expression.

We believe that: (1) CCK receptor antagonists may inhibit the positive regulation of the ligand by the downstream signaling pathway, thereby reducing the transcriptional level; (2) blocking CCK receptor reduces the sensitivity of EECs to CCK, indirectly affecting the autocrine or paracrine feedback regulation of CCK, thereby reducing the transcription of the CCK gene. Importantly, stimulation of cholinergic signaling markedly enhanced CCK secretion from EECs, which again could be abolished by selective blockers of TRPV4 channels, NCX1 and CCK receptors. Furthermore, these pharmacological blockers effectively ameliorated defecation volume and symptoms of diarrhea in IBS‐D mice, such as water content and number of fecal particles (Figure 9). These data obtained from the in vivo study are consistent with the data from the in vitro study in EECs, strongly suggest the involvement of the TRPV4/NCX1/Ca^2+^/CCK pathway in the development of IBS‐D, and therefore the molecules on this pathway may be potential novel targets for the prevention/treatment of IBS‐D. Moreover, based on the current theory that vagus nerves modulates enterohormone secretion from EECs via cholinergic signaling to constitutes a pivotal component of the brain–gut axis (Bonaz et al., 2018), we reveal a novel molecular mechanism in this axis that is likely involved in the pathogenesis of IBS‐D. However, given the multifactorial nature of IBS‐D pathophysiology, it is possible that changes in other enteroendocrine hormones, such as GLP‐1 and PYY, may also contribute to the observed in vivo phenotypes. In addition, since TRPV4 and NCX1 are not only expressed in EECs but also in various other cell types within the gastrointestinal tract, future cell‐type‐specific analyses would further clarify the role of the TRPV4/NCX1 pathway in IBS‐D.

## AUTHOR CONTRIBUTIONS

**Jianxin Wang:** Data curation; formal analysis; investigation. **Xiongying Chen:** Data curation; investigation. **Shuo Tong:** Data curation; investigation. **Chengxin Qu:** Data curation; investigation. **Wei Lu:** Methodology; supervision. **Hui Dong:** Conceptualization; funding acquisition; methodology; supervision.

## FUNDING INFORMATION

These studies were supported by research grants from the National Natural Science Foundation of China (No. 82570647 and No. 82273115 to H.D.).

## CONFLICT OF INTEREST STATEMENT

The authors declare that they have no conflicts of interest with the contents of this article.

## ETHICS STATEMENT

The animal study was reviewed and approved by the Ethics Committee at the Medical College of Qingdao University.

## Data Availability

Data will be made available on request.
